# The characterisation of *Wickerhamomyces anomalus* M15, a highly tolerant yeast for bioethanol production using seaweed derived medium

**DOI:** 10.3389/fbioe.2022.1028185

**Published:** 2022-10-13

**Authors:** William Turner, Darren Greetham, Chenyu Du

**Affiliations:** ^1^ School of Applied Science, University of Huddersfield, Huddersfield, United Kingdom; ^2^ Department of Molecular Biology and Biotechnology, University of Sheffield, Sheffield, United Kingdom

**Keywords:** Marine yeast, Substrate and product inhibitory affect, Ethanol tolerance, *Porphyra umbilicalis*, *Ulva linza* and *Laminaria digitata*, Macroalgae

## Abstract

Advanced generation biofuels have potential for replacing fossil fuels as society moves forward into a net-zero carbon future. Marine biomass is a promising source of fermentable sugars for fermentative bioethanol production; however the medium derived from seaweed hydrolysis contains various inhibitors, such as salts that affected ethanol fermentation efficiency. In this study the stress tolerance of a marine yeast, *Wickerhamomyces anomalus* M15 was characterised. Specific growth rate analysis results showed that *Wickerhamomyces anomalus* M15 could tolerate up to 600 g/L glucose, 150 g/L xylose and 250 g/L ethanol, respectively. Using simulated concentrated seaweed hydrolysates, *W. anomalus* M15’s bioethanol production potential using macroalgae derived feedstocks was assessed, in which 5.8, 45.0, and 19.9 g/L ethanol was produced from brown (*Laminaria digitata*), green (*Ulva linza*) and red seaweed (*Porphyra umbilicalis*) based media. The fermentation of actual *Ulva spp.* hydrolysate harvested from United Kingdom shores resulted in a relatively low ethanol concentration (15.5 g/L) due to challenges that arose from concentrating the seaweed hydrolysate. However, fed-batch fermentation using simulated concentrated green seaweed hydrolysate achieved a concentration of 73 g/L ethanol in fermentations using both seawater and reverse osmosis water. Further fermentations conducted with an adaptive strain *W. anomalus* M15-500A showed improved bioethanol production of 92.7 g/L ethanol from 200 g/L glucose and reduced lag time from 93 h to 24 h in fermentation with an initial glucose concentration of 500 g/L. The results indicated that strains *W. anomalus* M15 and *W. anomalus* M15-500A have great potential for industrial bioethanol production using marine biomass derived feedstocks. It also suggested that if a concentrated high sugar content seaweed hydrolysate could be obtained, the bioethanol concentration could achieve 90 g/L or above, exceeding the minimum industrial production threshold.

## 1 Introduction

The recent soar in energy prices across Europe has sparked another round of exploration for commercially viable alternative energy sources, including biofuels. Biofuels, which can be produced from crops, agriculture residues, municipal solid waste and other biomass, are an ideal candidate to meet the increasing energy demand. Not only are they essential in the move towards carbon neutrality and reducing the polluting effects of fossil fuels; biofuels are the only sustainable energy source that can utilise existing transportation fuel distribution infrastructure and existing vehicles. Biofuel is particularly beneficial to rural economics. More importantly, developing biofuels would improve national energy security, reducing Europe’s dependence of energy importation from increasing unstable fossil fuel markets.

Algal biofuel is generally considered as an advanced generation of biofuel; it does not use arable land for feedstock production and does not compete with food and feed applications of biomass, therefore it is expected to be more sustainable than first and second generation biofuels (Jambo et al., 2016). Marine macroalgae biomass grows in abundance globally. Although cultivation of seaweed has not been widely investigated apart from south Asian region, worldwide macroalgae production already reached 34.5 million tonnes dry weight annually, indicating it is a suitable renewable biomass for biofuel production (Saji et al., 2022).

All three major types of Macroalgae, brown seaweed (*Phaeophyta*), green seaweed (*Chlorophyta*) and red algae (*Rhodophyta*) have been explored for bioethanol production (Kostas et al., 2016; Greetham et al., 2020). In a typical bioethanol fermentation process, seaweed is initially pre-treated with dilute acid or alkali (Kumar et al., 2013; Fernand et al., 2017) at elevated temperature to form a sugar rich seaweed hydrolysate. Then, the hydrolysate is fermented to bioethanol by yeasts e. g. *Saccharomyces* spp. *Pichia angophorae* (Horn et al., 2000) and *Defluviitalea phaphyphila* (Ji et al., 2016) or recombinant *Escherichia coli* strain (*E. coli* KO11, Wargacki et al., 2012). One of the challenges in seaweed based bioethanol production is low ethanol concentration by the end of the fermentation, which is typically in the range of 20–30 g/L (Greetham et al., 2018). By comparison, in an industrial bioethanol production process, a higher ethanol concentration is expected (e.g. 80–150 g/L) to reduce the cost of distillation (Du et al., 2016).

In order to improve ethanol titer, and consequently the economic feasibility of an algal biofuel process, various strategies have been explored to improve hydrolysis yield to liberate higher amount of sugars, such as successive enzymatic saccharification (Yanagisawa et al., 2011), combined physiochemical and enzymatic hydrolysis process (Adams et al., 2011), simultaneously saccharification and fermentation (SSF, Hargreaves, et al., 2013) and hydrolysis with seawater instead of fresh water (Greetham et al., 2020). For the successive saccharification process, Yanagisawa et al., 2011 obtained media containing 78.8 and 123 g/L glucose from green weed (*Ulva pertusa*) and brown seaweed (*Alaria crassifolia*), respectively, achieving ethanol production of 30.0 and 34.4 g/L respectively. The research group also obtained a red seaweed hydrolysate (*Gelidium elegans*) using combined physiochemical and enzymatic hydrolysis process, which contained 70.9 g/L glucose and 53.2 g/L galactose. 55.0 g/L ethanol was produced in 72 h during which both glucose and galactose were consumed (Yanagisawa et al., 2011). In a study carried out by Hargreaves, et al., 2013, a hydrolysate containing 63.2 g/L galactose was obtained from red seaweed *Kappaphycus alvarezii*, which was then used in a SSF process containing the seaweed derived residue cellulose (18% w/v). After 6 days of fermentation using strain *Saccharomyces cerevisiae* CBS178, 65 g/L bioethanol was obtained. This is one of the highest bioethanol concentrations achieved using seaweed-derived media. However, it was noticed that around 20 g/L glucose and galactose remained at the end of the fermentation, the authors proposed ethanol tolerance could have hampered further ethanol synthesis (Hargreaves, et al., 2013).

Alternatively, the concentration of sugars in the seaweed hydrolysate could be enhanced after saccharification step, e.g. *via* rotary evaporation (Ge et al., 2011; Greetham et al., 2020). Greetham et al., 2020 concentrated a green seaweed hydrolysate (*Ulva linza*) to a total sugar content of ∼350 g/L, which was then diluted and used for fed-batch fermentation, producing 48.2 g/L ethanol. One of the drawbacks of this method is that the salt and inhibitory contents in the hydrolysate will be concentrated as well, which could inhibit the cell growth and ethanol synthesis. Therefore, a robust strain with high salt and inhibitor tolerance is preferred, such as using marine yeasts for the bioethanol production (Zaky et al., 2018).

As discussed above, one of key challenges of further developing algal biofuels is the development of a strain that tolerates high stress during the fermentation and is capable of ethanol synthesis with high efficiency. In this study, characterisation of a highly halotolerant marine yeast (*Wickerhamomyces anomalus* M15) for its sugar and ethanol tolerance was initially explored. Then the bioethanol production potential using simulated concentrated brown, green and red seaweed hydrolysate for bioethanol fermentation was carried out. Next actual green seaweed hydrolysate (mixed *Ulva spp.*) was concentrated and used for bioethanol fermentation. As the sugar content in the seaweed hydrolysate was low, simulated concentrated green seaweed hydrolysate was further used to investigate the bioethanol production potential of the strain and its derivative in batch and fed-batch fermentations to maximise ethanol production.

## 2 Materials and methods

### 2.1 Microorganism

W*ickerhamomyces anomalus* M15 is a marine yeast strain isolated from marine debris collected from the United Kingdom coastline. The yeast was preserved and maintained on YPD medium (20 g/L glucose, 20 g/L peptone and 10 g/L yeast extract). W*ickerhamomyces anomalus* M15-500A is an adaption strain of W*. anomalus* M15 which was preserved after 790 h of fermentation using 500 g/L glucose, 20 g/L peptone and 10 g/L yeast extract.

### 2.2 Marine yeast tolerance to fermentation substrates, products and inhibitors

The cell growth profile of *W. anomalus* M15 was assessed using a BMG Labtech SPECTROstar Nano plate reader (Corning Costar flat bottomed 96 well plate) to determine its tolerance to glucose, xylose, arabinose, ethanol, sodium chloride and sodium acetate. These chemicals used are either a substrate or common metabolite in a typical fermentation process. Sodium chloride was also included to test the tolerance range of the marine yeast *W. anomalus* M15. A range of concentrations (as shown in Table 1) were used to determine toxicity by analysing the change in specific growth rate, and lag time.

**TABLE 1:** Sugar compositions in the simulated brown, green and red seaweed hydrolysates.

	Glucose (G/L)	Xylose (G/L)	Arabinose (G/L)	Galactose (G/L)	Mannitol (G/L)	Total sugars (G/L)
*L. digitata*						
Natural	1.32	0.62	0.16	0.55	17.49	20.14
5x	6.6	3.1	0.8	2.75	87.45	100.7
7.5x	9.9	4.65	1.2	4.13	131.2	151.1
10x	13.2	6.2	1.6	5.5	174.9	201.4
*U. linza*						
Natural	8.16	5.51	2.27	0.67	—	16.61
5x	40.8	27.55	11.35	3.35	—	83.05
7.5x	61.2	41.33	17.03	5.03	—	124.6
10x	81.6	55.1	22.7	6.7	—	166.1
*P. umbilicalis*						
Natural	3.52	2.95	0.32	6.29	—	13.08
5x	17.6	14.75	1.6	31.45	—	65.40
7.5x	26.4	22.13	2.4	47.18	—	98.10
10x	35.2	29.5	3.2	62.9	—	130.8

An overnight culture of *W. anomalus* M15 was grown in YPD medium and used to inoculate each well at an optical density (OD_600_) of 0.1 at the start of the experiment.

The media used to fill each well (100 µL) was prepared from autoclaved (121°C, 20 min) concentrated stocks of each component (as shown in Table 1) aliquoted in varying quantities into each well and made up to 100 µL (including inoculum) with filtered sterilised deionised water to produce the range of concentrations required. All experiments were carried out in triplicate. Control wells were filled with YPD medium only (not inoculated). The plate reader measured optical density at OD_600_ every 30 min. Plates were incubated statically at 30°C for up to 90 h.

For the analysis of the impact of sugars (glucose, xylose and arabinose), the media was composed of peptone (20 g/L) and yeast extract (10 g/L) and a sugar: glucose (0–250 g/L), arabinose (0–100 g/L) and xylose (0–100 g/L). Tolerance of *W. anomalus* M15 to ethanol was measured with a range of 0–210 g/L, in YPD media.

### 2.3 *W. anomalus* M15 fermentation of synthetic seaweed hydrolysate

Simulated green, brown and red seaweed hydrolysates were prepared for the exploration of bioethanol fermentation potential. The compositions of the natural semi-synthetic hydrolysates were the same as reported previously (Greetham et al., 2020, Table 1). As the initial seaweed hydrolysate contained a low sugar content, a range of semi-synthetic media containing 5x, 7.5x and 10x concentrated sugars of initial seaweed hydrolysates were prepared, as shown in Table 1. Fucose and rhamnose were omitted due to their relative low quantities.

Media was autoclaved (121°C, 20 min) and transferred into Wheaton bottles in triplicate aliquots (100 ml). A magnetic flea was added, and the media was inoculated with *W. anomalus* M15 yeast to achieve an optical density (OD_600_) of 1.0. It was then sealed with a septum and foil cap. A needle was placed through the septum to allow the dispersal of CO_2_. The Wheaton bottles were placed on a magnetic stirring plate at 25°C. Weight loss was recorded regularly. At the end of the experiment, samples of 1 ml were taken, centrifuged (9,000 x g, 5 min), filtered (0.22 µm PTFE filter, polytetrafluoroethylene) and frozen for HPLC/GC analysis.

### 2.4 Fed-batch fermentation of semi-synthetic green seaweed hydrolysate

Fed-batch fermentations of semi-synthetic green seaweed hydrolysate were carried out. The starting media contained 5x concentrated sugars of *U. linza* hydrolysate as described in Table 1 plus yeast extract 10 g/L and peptone 20 g/L. The media were prepared using deionised water (DW) and seawater (SW), respectively. Two sugar syrups were prepared in a 50x concentration of the natural sugar content using deionised water and seawater (both filter sterilised). Media (20 ml) was added to Wheaton bottles (30 ml) and inoculated with *W anomalus* M15 to an OD_600_ of 0.5 from an overnight culture. Fermentations were incubated at 30°C and agitated at 300 rpm using a magnetic stirrer bar. Weight loss was recorded three times daily and a sample (2 ml) was taken every 24 h. Sugar syrup additions (2 ml) were added after each sample was taken, retaining batch volume of 20 ml.

### 2.5 Green seaweed collection and processing

Green seaweed was harvested from two locations on the United Kingdom coastline (Rhosneigr and Wallasey beach) following the Detailed Guidance for Seaweed Harvesting—Hand Gathering guidance note provided by Natural Resources Wales (Cdn.naturalresources.wales, 2022). Green seaweed was collected at low tide; small amounts of loose washed seaweed were picked up, as well as seaweed harvested, by cutting the weed above the holdfast with scissors.

Approximately 1 kg of wet seaweed was collected from each location. The seaweed was washed in freshwater to remove sand, debris and other seaweed species through flotation and manual separation. All seaweeds collected and used were varying *Ulva* species. The seaweed was pressed in a sieve to remove excess water, placed in a resealable bag and subsequently frozen at −20°C.

The frozen seaweed was subjected to freeze drying for 72 h. Once dry, the seaweed was milled by rubbing through a 1 mm screen sieve. Dried, milled seaweed was stored until further use.

### 2.6 Seaweed pre-treatment and concentration

50 g dry seaweed was added to seawater and 5% H_2_SO_4_ at a 1:10 solid load ratio and autoclaved at 121 °C for 15 min. Following pre-treatment, the hydrolysate was filtered through a muslin cloth using a Buchner funnel and vacuum to remove solid biomass and neutralised using NaOH. The hydrolysate was further filtered to using a Buchner funnel and Whatman filter paper to clarify.

The seaweed hydrolysate was subsequently concentrated by heating to approximately 80°C on a hot plate. The hydrolysate was concentrated to around 3–4x its original volume (450 ml–∼120 ml), at which point salt saturation was beginning to occur. Cooling to 4 °C caused salt to crystallise. To remove crystallised salt from the hydrolysate, the cooled solution was filtered using a 0.22 µm bottle top filter. The salinity of the filtered hydrolysate was measured using a Thermo Fisher Eutech Elite CTS probe, to determine that the salt content was approximately 140 g/L, therefore too saline for yeast growth.

### 2.7 Ion exchange

Due to the excess salt content, the seaweed hydrolysate was filtered through Na and Cl ion exchange resins (AmberLite™ IRC120 Na and AmberLite™ IRA402 Cl). Resins were regenerated using an acid/base (HCl 1M for Na, NaOH 1M for Cl) stirred for 1 h, the washed with ultrapure water until pH6. Excess water was removed, then 220 cm^3^ of each resin was placed in a column and seaweed hydrolysate was filtered through. The hydrolysate salinity was measured after each pass of both resins using the CTS probe, until all hydrolysates were approximately at 60 g/L salt content.

### 2.8 Fermentation of concentrated green seaweed hydrolysate

Mini fermentation vessels were prepared by the addition of a magnetic flea to a Wheaton bottle (30 ml), and sterilised by autoclaving, along with septa. Reverse Osmosis (RO) water and seawater-based seaweed hydrolysates were transferred into Wheaton bottles in duplicate aliquots (27 ml). A concentrated solution of yeast extract and peptone (100 g/L, 200 g/L) was inoculated with *W. anomalus* M15 and added to the hydrolysate (3 ml) to generate a total solution volume of 30 ml and starting optical density of 0.5. Starting pH was 5 and solution salinity was approximately 60 g/L. The mini fermentation vessels were then sealed with a septum and crimped foil cap, pierce with a sterile needle to allow dispersal of CO_2_. The Wheaton bottles were placed on a magnetic stirring plate (350 rpm) in a static incubator (30°C). Weight loss was recorded 4 times daily (weekdays). Sampling (0.5 ml), optical density and pH were recorded once daily. Samples were centrifuged (9,000 × g, 5 min) frozen for later ethanol analysis. Samples were diluted and filtered (0.22 µm PTFE filter) prior to HPLC/GC analysis.

### 2.9 High gravity fermentation using *W. anomalus* M15 and *W. anomalus* M15-500A

In high gravity fermentations, YPD media prepared with enhanced glucose concentrations of 100, 200 and 300 g/L were used for fermentations with *W. anomalus* M15. For the fermentations with *W. anomalus* M15-500A, YPD media with enhanced glucose concentrations of 100, 200 and 500 g/L glucose were used. The fermentation was carried out using Wheaton bottle (30 ml) fermenters following conditions described in section 2.8.

### 2.10 ethanol analysis using gas chromatography flame ionization detection (GC-FID)

Ethanol analysis of fermentation samples was conducted on an Agilent 6890 GC-FID with a Supelco SPB-5 column. The injector and detector were set to 250°C and 300°C respectively. Helium was used as the carrier gas at a flow rate of 1 ml/min. A 1 µL manual injection was used to inject each sample, splitless. The oven was set to run for 10 min at a rate of 20°C/min from 60 to 200°C after an initial delay of 2 min.

Before injection each sample was centrifuged (9,000 × g, 5 min), filtered through a 0.22 µm PTFE filter and diluted by a factor of 10 or 100 depending on concentration.

### 2.11 Sugar analysis using HPLC (high performance liquid chromatography)

Sugar analysis of hydrolysates was conducted using a Dionex ICS3000 HPAEC with a ThermoFisher CarboPac PA20 3 mm × 150 mm column. 10 and 200 mm NaOH mobile phase was used; fucose, arabinose, glucose, galactose and xylose were used for standards. Standards were prepared in a 10–100 PPM concentration range. Samples were diluted by a factor of 500 and 2000 to an expected concentration of 10–50ppm and filtered through a 0.22 µm PTFE filter.

## 3 Results

### 3.1 Marine yeast characterisation

**FIGURE 1:**
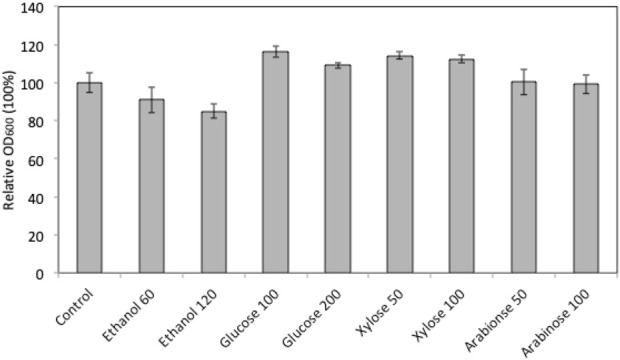
Relative cell growth of *W. anomalus* M15 in fermentation with the addition of sugars or ethanol. Control, YPD medium; ethanol 60, 60 g/L ethanol; ethanol 120, 120 g/L ethanol; Glucose 100, 100 g/L glucose; Glucose 200,200 g/L glucose; Xylose 50, 50 g/L xylose; Xylose 100, 10 g/L xylose; Arabinose 50, 50 g/L arabinose; Arabinose 100, 100 g/L arabinose.

In order to explore the potential of using marine yeast *W. anomalus* M15 in high gravity fermentation, the strain’s tolerance to ethanol, glucose, xylose and arabinose was characterised using a microplate reader growth assay. Figure 1 compares the cell growth of *W. anomalus* M15 after 90 h cultivation in media containing different concentrations of ethanol and glucose, xylose and arabinose. In fermentations containing extra glucose and xylose, cell growth improved. Cell growth was impaired by the addition of ethanol at 60 and 120 g/L concentration in comparison to the control. The addition of arabinose at two different concentrations did not appear to have any impact on cell growth. The impact of glucose, xylose and ethanol on the cell growth of *W. anomalus* M15 in wider ranges was subsequently investigated and the cell growth curve was monitored. The impact of arabinose was not included, as it does not facilitate or inhibit cell growth.

#### 3.1.1 *W. anomalus* M15 tolerance to glucose

**FIGURE 2:**
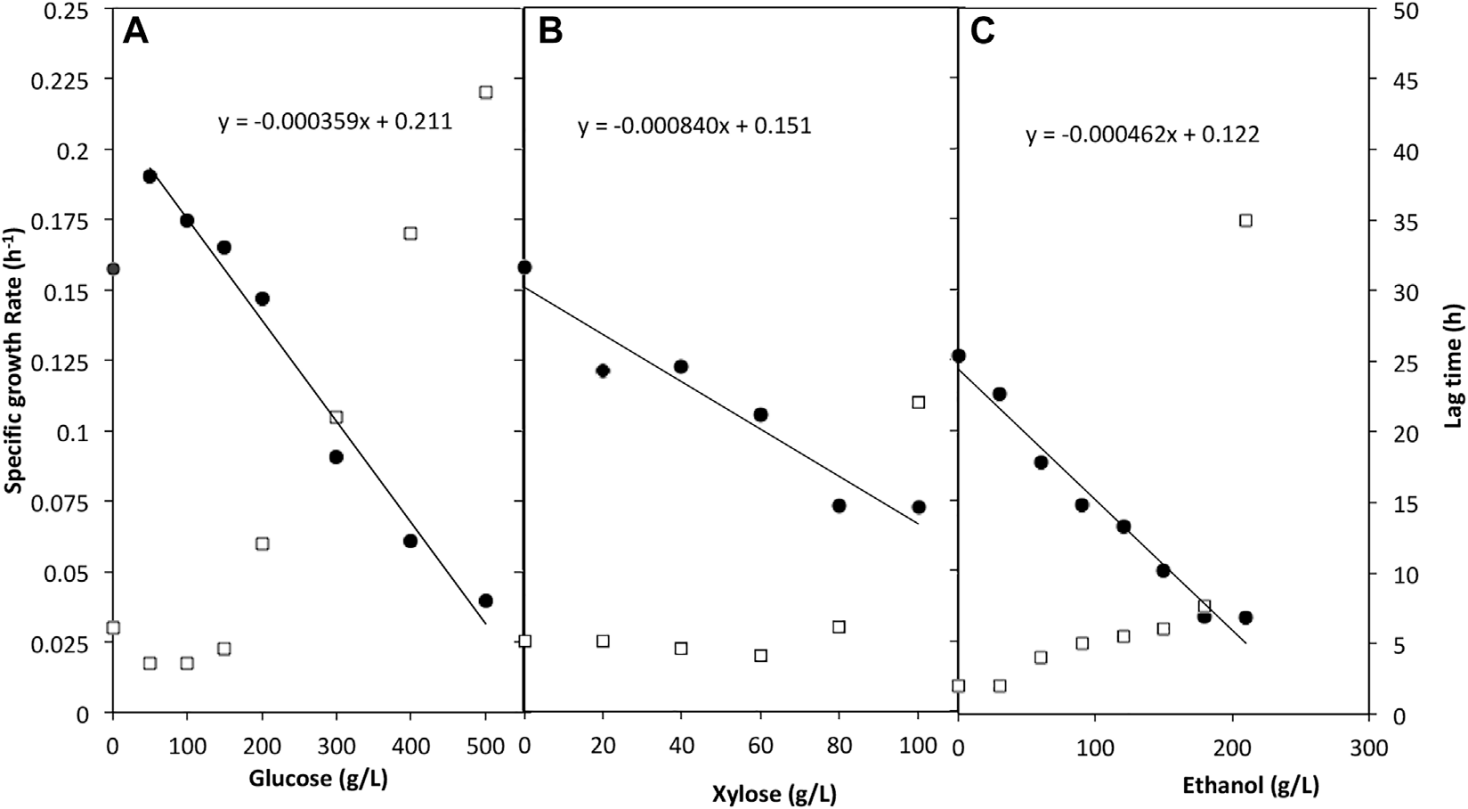
The impact of glucose **(A)** xylose **(B)** and ethanol **(C)** on the specific growth rate and lag time of *W. anomalus* M15. Solid filled circle: specific growth rate; open square: lag time.

For the tolerance to glucose, a wide concentration range of 0–500 g/L was assessed. *W. anomalus* M15 tolerated all concentrations of glucose measured. The whole cell growth profiles were presented in Supplementary Appendix Figure AS1, while the specific cell growth rate and lag time were shown in Figure 2A. The presence of glucose improved the specific growth rate for fermentations with initial glucose concentrations lower than 200 g/L compared to control (yeast extract 10 g/L, peptone 20 g/L, no glucose). The lag time preceding growth phase was reduced in media containing glucose for the same trials. Samples containing 200–500 g/L glucose had a considerably higher lag time from 12 h to 44 h. In the control where no glucose was added, the cell can still grow using only yeast extract and peptone. As shown in the trend line in Figure 2A, *W. anomalus* M15 could potentially tolerate a glucose concentration as high as near 600 g/L, although lag time may be considerably affected, impeding fermentation.

#### 3.1.2 *W. anomalus* M15 tolerance to xylose

A range of 0–100 g/L xylose was used to determine utilisation of and tolerance to xylose by *W. anomalus* M15 (Figure 2B). It proved that *W. anomalus* M15 tolerated xylose throughout the concentration range used, and lag time decreased marginally as the concentration increased up to a concentration of 60 g/L xylose, however the specific growth rate decreased continuously. As the concentration increased after 60 g/L, lag time increased sharply. But when the microorganism’s growth started, cells can reach similar final cell density (Supplementary Appendix Figure AS2). The trend line simulation suggested that *W. anomalus* M15 could tolerate up to 150 g/L xylose.

#### 3.1.3 *W. anomalus* M15 tolerance to ethanol

Cell growth studies using plate reader were carried out in a range of ethanol concentrations between 0 and 210 g/L. The whole cell growth profiles were presented in Supplementary Appendix Figure AS3. Results proved that *W. anomalus* M15 can tolerate ethanol at all ranges of ethanol measured (Figure 2C). The specific growth rate data shows that although the strain tolerated ethanol in the media, increase in the ethanol concentration had a pronounced effect on the growth rate. Lag time also escalated along with the increase in ethanol concentration, especially at a concentration of 210 g/L. The trend line shows in Figure 2C suggested that *W. anomalus* M15 could tolerate up to approximately 250 g/L ethanol before total cell death.

### 3.2 *W. anomalus* M15 fermentation using concentrated semi-synthetic seaweed hydrolysates

The tolerance experiment indicated that strain *W. anomalus* M15 could potentially tolerate 600 g/L glucose, 150 g/L xylose and 250 g/L ethanol, respectively, suggesting it is an excellent candidate for use in high gravity bioethanol fermentation. In a previous study, in seaweed hydrolysis with a solid loading ratio of 10% (w/v), the total sugar contents obtained were 20.1, 16.6 and 13.1 g/L, containing 1.32, 8.16, and 3.52 g/L glucose for brown, green and red seaweeds, respectively (Greetham et al., 2020, Table 1). The sugar contents are far from the tolerant limits of the strain *W. anomalus* M15. In order to explore the bioethanol production potential, concentrated semi-synthetic seaweed hydrolysate (up to 10 times concentrated seaweed hydrolysate, designated 10x) were used for fermentations with *W. anomalus* M15. For 1x synthetic media, after 144 h fermentations, 1.13, 4.26, and 3.39 g/L ethanol were obtained, for brown, green and red seaweed respectively (Table 2). It can be seen that for ethanol yield based on glucose, most of the results are higher than 100% of the theoretic yield, indicating other sugars have been used for bioethanol production. For yield calculated based on both glucose and galactose, fermentations using brown seaweed based semi-synthetic media still showed a yield over 100%, suggesting besides glucose and galactose, other sugars, xylose, arabinose or mannitol have been used for ethanol synthesis by *W. anomalus* M15. Early experiments have demonstrated that although *W. anomalus* M15 can grow in a medium containing only yeast extract and peptone (Supplementary Appendix Figure AS1), production of ethanol was not detected (Data not shown).

**TABLE 2:** ethanol productions in fermentations using natural and concentrated semi-synthetic seaweed hydrolysate media.

	Glucose (g/L)	Glucose + galactose (g/L)	Total sugar (g/L)	Ethanol (g/L)	Yield based on glucose (%)	Yield based on glucose + galactose	Yield based on total sugar (%)
*L. digitata*							
YPD	20	20	20	9.8 ± 2.03	95.7	95.7%	95.7
Natural	1.32	1.87	20.14	1.13 ± 0.04	167.2	118.0%	11.0
5x	6.6	9.35	100.7	5.41 ± 0.97	160.1	113.0%	10.5
7.5x	9.9	14.03	151.1	7.87 ± 2.19	155.3	109.6%	10.2
10x	13.2	18.7	201.4	5.79 ± 1.37	85.7	60.5%	5.6
*U. linza*							
YPD	20	20	20	10.03 ± 0.52	97.9	97.9%	97.9
Natural	8.16	8.83	16.61	4.26 ± 0.48	102.0	94.2%	50.1
5x	40.8	44.15	83.05	20.43 ± 2.80	97.8	90.4%	48.0
7.5x	61.2	66.23	124.6	34.7 ± 4.40	110.7	102.3%	54.4
10x	81.6	88.3	166.1	45.04 ± 4.40	107.8	99.6%	53.0
*P. umbilicalis*							
YPD	20	20	20	10.31 ± 1.36	100.7	100.7%	100.7
Natural	3.52	9.81	13.08	3.39 ± 0.28	188.1	67.5%	50.6
5x	17.6	49.05	65.4	7.49 ± 2.26	83.1	29.8%	22.4
7.5x	26.4	73.58	98.1	13.61 ± 0.37	100.7	36.1%	27.1
10x	35.2	98.1	130.8	19.85 ± 2.64	110.1	39.5%	29.6

**FIGURE 3:**
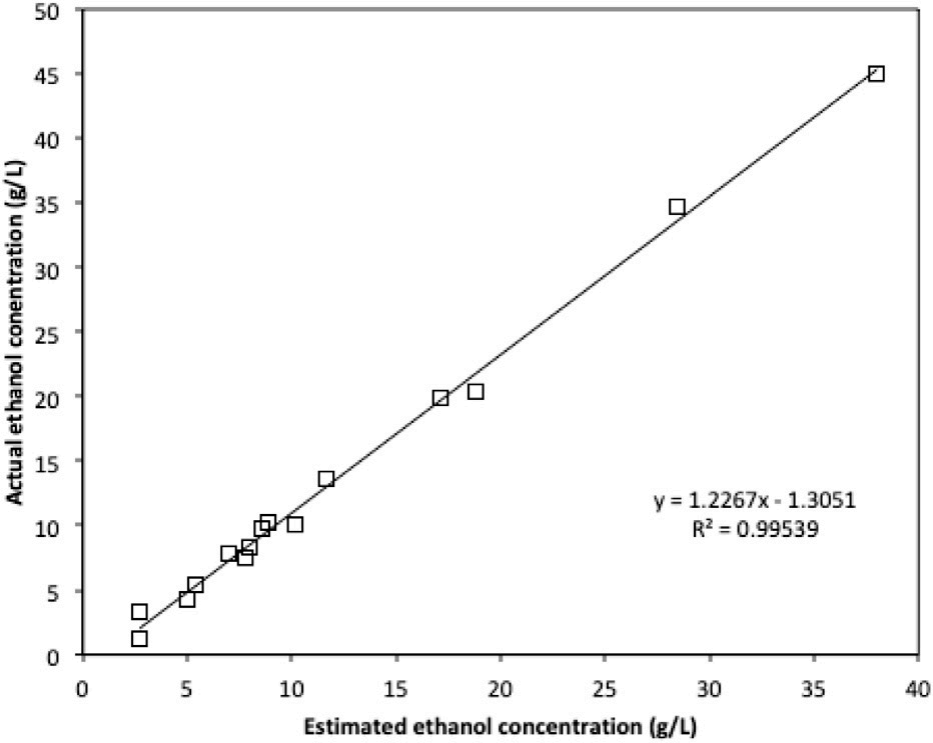
The comparison of ethanol concentration obtained by GC-FID and the estimated ethanol concentration based on weight loss.

**FIGURE 4:**
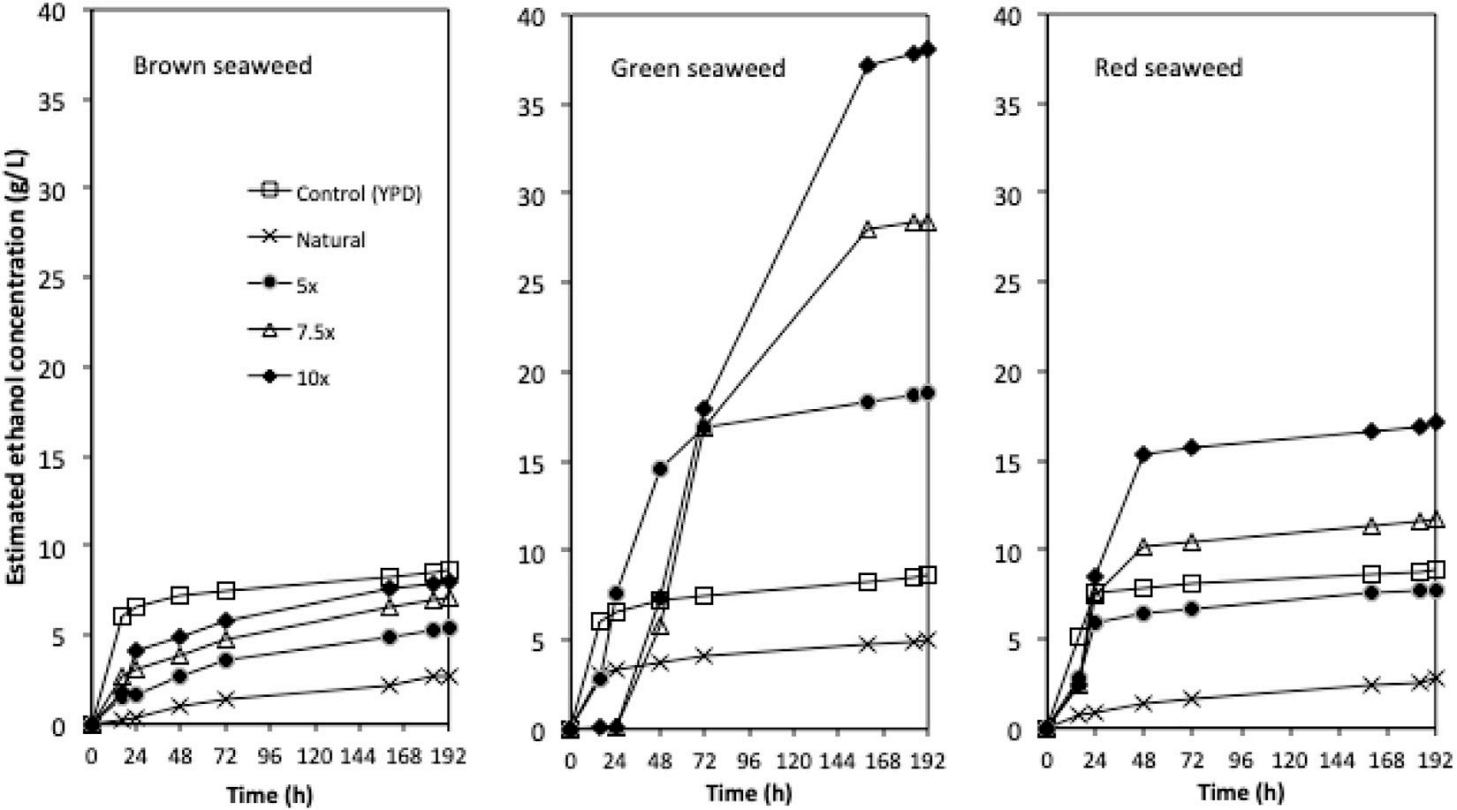
The ethanol concentration profiles for fermentations using concentrated semi-synthetic brown seaweed, green seaweed and red seaweed media. 5x: 5-times concentrated hydrolysate; 7.5x: 7.5-times concentrated hydrolysate; 10x: 10-times concentrated hydrolysate.

Due to the limited access of GC-FID, weight loss data was monitored throughout the fermentation and used to determine the ethanol production profiles. The ethanol concentration was estimated based on the weight loss data using the method described in the Method section. The final samples at the end of each experiment were analysed using GC-FID and the actual ethanol concentrations were compared with estimated ethanol concentrations (Figure 3). Ethanol concentrations estimated from weight loss showed a linear relationship with the actual measured ethanol concentrations. Therefore, the estimated ethanol concentrations were used to present the ethanol accumulation profiles in the experiments (Figure 4). For fermentations using synthetic brown seaweed and red seaweed media, most of ethanol was synthesized within 48 h, which probably correlated with the depletion of glucose in the fermentation as observed in previous fermentation (Greetham et al., 2020). The small increase in ethanol concentrations after 48 h could relate to galactose utilization. For fermentation using 10x concentrated synthetic *L. digitata* hydrolysate, only 5.79 g/L ethanol was obtained with a yield of 60.5% of the theoretical yield (Table 2). This was lower than that obtained in fermentation using 7.5x concentrated synthetic *L. digitata* hydrolysate. This is probably due to the high mannitol concentration (171 g/L) in the fermentation media, which may inhibit the cell growth and ethanol synthesis. For experiments using synthetic green seaweed hydrolysate, the fermentation took longer to reach stationary stage. As obtained in tolerance experiment, the range of initial sugar concentrations (glucose up to 81.6 g/L, xylose up to 55.1 g/L, arabinose 22.7 g/L, galactose 62.9 g/L) did not show an inhibitory affect on *W. anomalus* M15 growth.

### 3.3 *W. anomalus* M15 fermentation using actual green seaweed hydrolysate

The fermentations using concentrated synthetic seaweed hydrolysate suggested U. linza would be most ideal macroalgae for bioethanol conversion. Fermentations using actual green seaweed hydrolysate were carried out. The sugar content in the hydrolysate is shown in Table 3. In the hydrolysate obtained using deionised water, approximately 29 g/L total sugar was achieved with 11 g/L glucose. In the hydrolysate obtained using seawater, approximately 40 g/L total sugar was achieved with ∼16 g/L glucose (Table 3). The concentration of the hydrolysates using simple evaporation method was attempted. By reducing the volume to around 30% of the original volume, the sugar contents in the hydrolysate were enhanced to 70.6 g/L and 137 g/L for hydrolysis using deionised water and seawater, respectively. After the evaporation process, some salts crystallised (mainly sodium sulphate) when the concentrated hydrolysate cool down to room temperature. The analysis showed that the total salt content in the concentrated hydrolysate reached 191.5 g/L. Ion exchange was applied to reduce the salt content. After the ion exchange process, the sugar content in the hydrolysate obtained using deionised water reached 122.80 g/L, while the total salt content was reduced to 62.5 g/L. Surprisingly, in the hydrolysate obtained using seawater, the sugar content reduced from 137 g/L to 53.6 g/L, along with the reduction of total salt content from 191.5 g/L to 57.7 g/L.

**TABLE 3:** sugar content.

	Fucose	Arabinose	Galactose	Glucose	Xylose	Sum
Hydrolysis using deionised water						
Hydrolysate	0.86 ± 0.20	8.64 ± 1.32	2.82 ± 0.40	11.00 ± 1.66	6.21 ± 1.11	28.67
Concentrated	2.77 ± 0.67	20.62 ± 3.97	6.89 ± 1.76	25.69 ± 4.84	14.59 ± 3.93	70.56
After IE	5.10 ± 0.57	39.32 ± 2.56	10.33 ± 1.24	41.53 ± 3.65	26.52 ± 4.13	122.80
Hydrolysis using seawater						
Hydrolysate	0.80 ± 0.01	9.72 ± 0.19	2.74 ± 0.09	15.82 ± 1.11	10.65 ± 0.41	39.72
Concentrated	3.43 ± 0.21	39.46 ± 2.34	6.82 ± 0.37	53.91 ± 2.71	33.70 ± 2.84	137.33
After IE	1.40 ± 0.08	15.65 ± 1.72	2.39 ± 0.07	25.21 ± 1.37	8.93 ± 0.61	53.58

**FIGURE 5:**
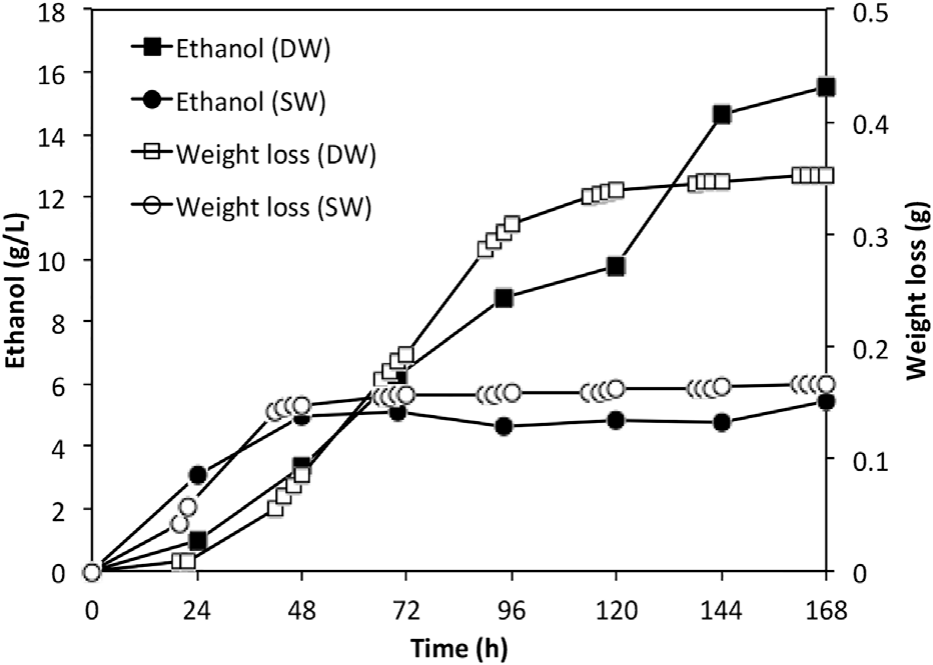
Ethanol profiles for fermentations using actual *Ulva* spp. hydrolysates. DW: deionized water; SW: sea water.

As the sugar concentrations were relatively low in the actual Ulva spp. hydrolysate, batch fermentation was carried out. The ethanol accumulation profiles were shown in Figure 5 along with the weight loss data. By the end of the fermentation, approximately 15.5 g/L and 5.45 g/L ethanol were achieved in fermentation using deionised water derived hydrolysate and seawater derived hydrolysate, respectively. The ethanol yields were 73.0% and 42.3% respectively, corresponding to an overall yield of 0.033 and 0.027 g ethanol per Gram dry weight seaweed.

### 3.4 High gravity fermentation using synthetic *U. linza* hydrolysate

**FIGURE 6:**
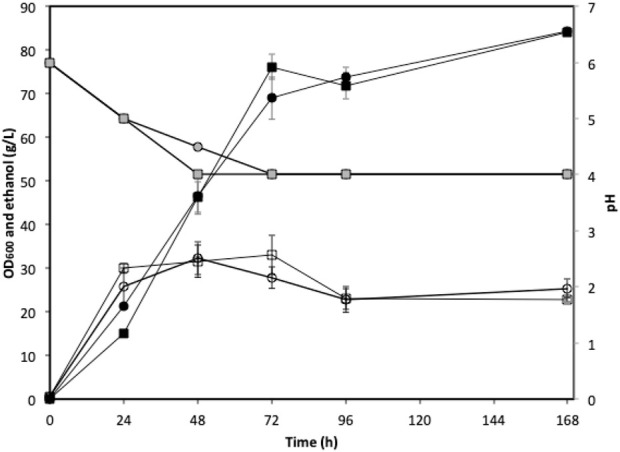
Fed-batch fermentation using green seaweed hydrolysate. Circle, deionized water; square, seawater; open symbols, OD_600_; black filled symbols: ethanol concentration; grey filled symbols: pH.

The low sugar content in the actual seaweed hydrolysate limited the exploration of the bioethanol production potential of strain W. anomalus M15, especially through fed-batch and high gravity fermentations. Therefore, synthetic U. linza hydrolysate was used in this section to explore the potential of the strain. Fed-batch fermentations were conducted using 5x concentrated synthetic *U. linza* hydrolysate prepared using either deionized water or seawater. The cell growth, ethanol production and pH profiles were indiscernible between fermentations using seawater and deionised water (Figure 6). After 96 h fermentation, the ethanol concentrations reached 71.8 ± 2.8 and 73.8 ± 4.8 g/L, for fermentations using seawater and deionised water, respectively. The average ethanol productivities at the 72 h were 1.06 and 0.96 g/L/h for fermentations using seawater and deionised water, respectively. The ethanol yields were 0.22 g/g total sugar in both cases. Along with the fermentation, the pH dropped from 6.0 to approximate 4.0.

Fermentations using extremely high glucose (500 and 750 g/L) glucose were carried out over a Christmas and Covid lockdown period. After 93 h of incubation, *W. anomalus* M15 grew up in 500 g/L samples; no cell growth was observed in fermentations using 750 g/L glucose (Supplementary Appendix Figure AS4). By the end of the experiment (790 h, after Christmas and Covid lock down), around 0.97 g/L ethanol was detected in the fermentation broth. The strains that survived long period of exposure to high glucose was isolated and designated as *W. anomalus* M15-500A.

**FIGURE 7:**
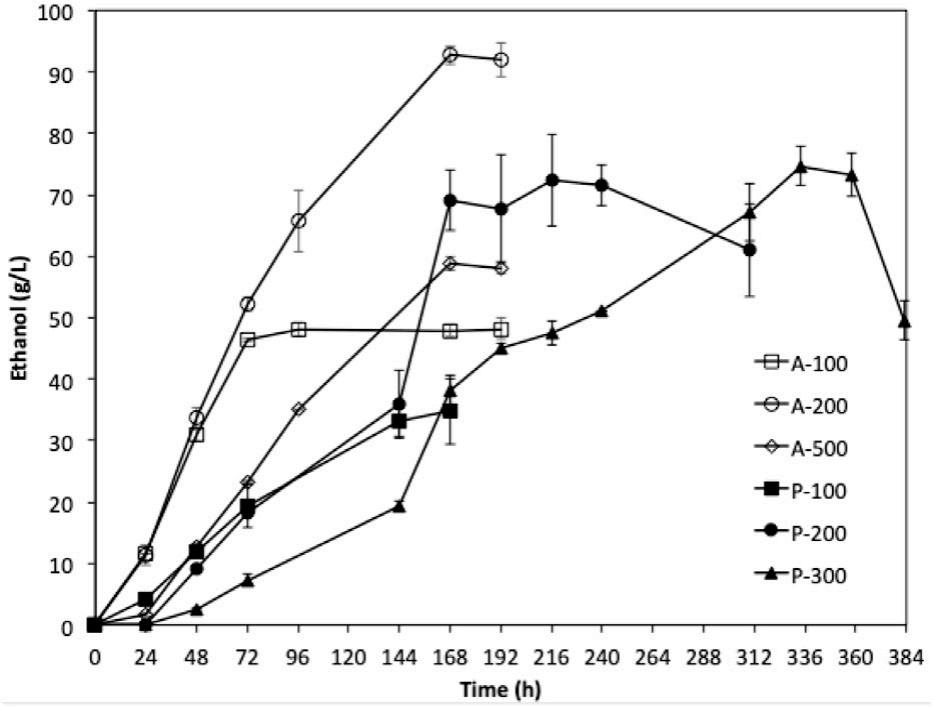
Fermentation of YPD media with different initial glucose concentration using *W. anomalus* M15 (P-100 100 g/L; P-200 200 g/L; P-300 300 g/L) and *W. anomalus* M15-500A (A-100 100 g/L; A-200 200 g/L; A-300 300 g/L).

Fermentations using the adaptive strain *W. anomalus* M15-500A were carried out using YPD media containing 100, 200, and 500 g/L glucose for 168 h. The results were compared with the fermentations by the parent strain using YPD media containing 100, 200, and 300 g/L glucose. As shown in Figure 7, the adaptive strain *W. anomalus* M15-500A consumed glucose significantly faster than the parent strain. For fermentation with 200 g/L glucose, 92.7 g/L ethanol was produced within 8 days, which is 28% higher than that of parent strain. More promisingly, the lag phase for fermentation of YPD medium containing 500 g/L significantly reduced from 93 h to less than 24 h.

## 4 Discussions

A robust and efficient microorganism is essential for a successful bio-production process. In a previous study, marine yeast *W. anomalus* M15 was found to possess a high tolerance to sodium chloride (21.4%), acetic acid (167.8 mmol/L) and furfural (49.6 mmol/L) (Greetham et al., 2019). With the aim of achieving a high ethanol concentration in a seaweed based biosynthesis process, the microorganism should be able to tolerate a high sugar content derived from seaweed and high final ethanol content. The results shown in Figure 2 suggested that strain *W. anomalus* M15 could tolerate up to 600 g/L glucose, which is 2-folds of the concentration (300 g/L) suggested by Caspeta et al., 2015 for an economic bioethanol production (Caspeta et al., 2015). Verification fermentations using *W. anomalus* M15 in 100 ml mini FVs were carried out with initial sugar content of 500 g/L and 750 g/L over a Covid lock down period. Cell growth was detected in the 500 g/L trial, but no cell growth in fermentation with 750 g/L (Supplementary Appendix Figure AS4). It is reported that excellent industrial bioethanol fermentation yeasts (*S. cerevisiae* PE-2 and CA1185) fermented a medium containing 335–343 g/L glucose, producing 151 g/L ethanol within 96 h (Pereira et al., 2011). The yeasts were used in 30% Brazil bioethanol plants producing 10% of bioethanol used worldwide. Comparing with *S. cerevisiae* PE-2 and CA1185, *W. anomalus* M15 could have better sugar tolerance and salt/inhibitor tolerance ability; although the ethanol productivity of *W. anomalus* M15 is not as good as these two industrial *S. cerevisiae* yeasts. The ethanol producing capacity of yeast could be improved by adaptation method (Dinh et al., 2008). An adaptation strain *W. anomalus* M15-500A was isolated from the fermentation broth by the end of the long period of fermentation using 500 g/L glucose. The adaptation strain showed significant improvement in sugar utilisation efficiency and bioethanol fermentation efficiency (92.7 g/L ethanol was produced within 168 h, Figure 7). The research showed strain *W. anomalus* M15-500A has a potential for industrial application in bioethanol production, especially using seaweed hydrolysate. Genome shuffling method could also be used to improve bioethanol production, which has been used by several researchers to generate mutant yeasts that were capable of fermenting media containing 300–320 g/L glucose, producing 120–150 g/L ethanol (Hou, 2010; Liu et al., 2011).

In order to investigate the bioethanol production potential of marine yeast *W. anomalus* M15, synthetic seaweed hydrolysate simulating brown seaweed (*L. digitata*), green seaweed (*U. linza*) and red seaweed (*P. umbilicalis*) were used for bioethanol fermentation. Concentrated synthetic seaweed hydrolysate up to 10x were also used to explore the behavior of *W. anomalus* M15 in fermentation of concentrated seaweed hydrolysate. As shown in Figure 4 and Table 2, synthetic green seaweed hydrolysate led to significant higher ethanol content, indicating green seaweed is a suitable substrate for bioethanol production, and preferred to brown and red seaweed when strain *W. anomalus* M15 was used. The results agreed with previous studies using the same strain with actual seaweed hydrolysate (Greetham et al., 2020). *Saccharomyces cerevisiae*, a strain closely related to *W. anomalus* M15 was tested for the fermentations of brown/green/red seaweed hydrolysates by Kostas et al., 2016, which also indicated that green seaweed would be a suitable substrate for bioethanol production. To enable utilization of sugars derived from brown and red seaweed, non-*Saccharomyces cerevisiae* strain could be used, such as *Pichia angophorae* (Horn et al., 2000) and *Defluviitalea phaphyphila* (Ji et al., 2016) for mannitol, algiante fermentation. Alternatively, construction of new metabolic pathways in model microorganisms, such as *S. cerevisiae* (Enquist-Newman et al., 2014) and *E. coli* (Wargacki et al., 2012) is a viable approach. However, it is challenging to construct a new pathway together with the installment of other desired properties for industrial biofuel fermentation, such as high stress tolerance. As a result, the ethanol concentrations in fermentation in these two studies were only 36–37 g/L (Wargacki et al., 2012; Enquist-Newman et al., 2014).

**FIGURE 8:**
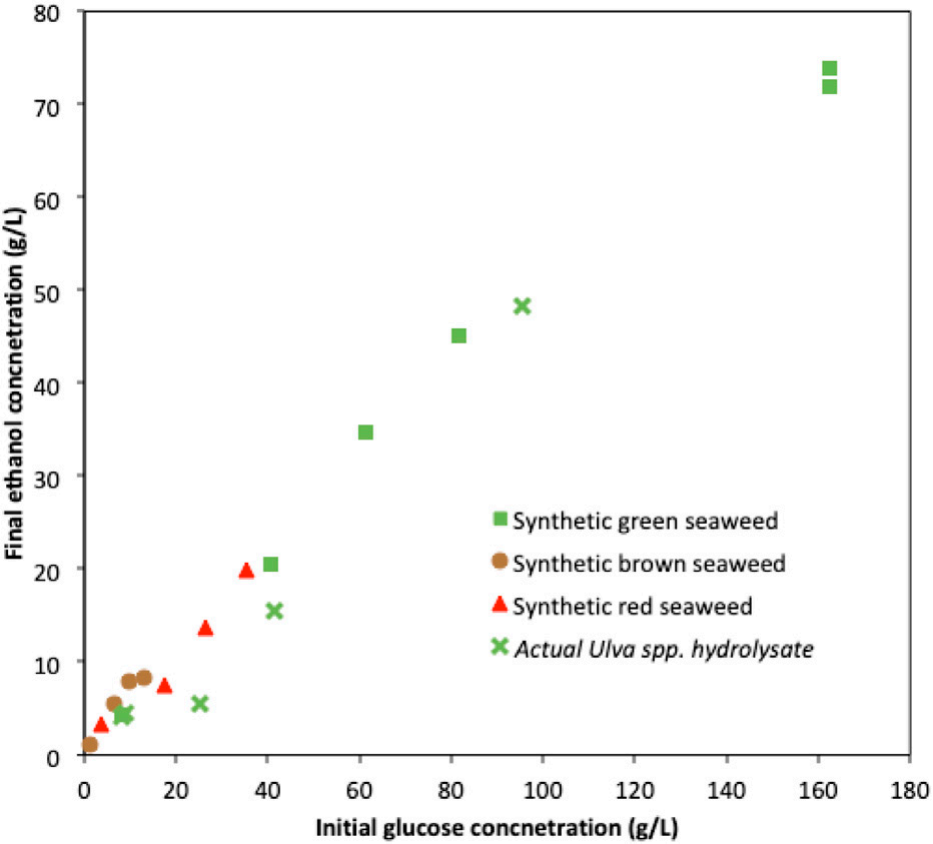
The relationship between initial glucose concentration in the seaweed and the final ethanol concentration after fermentation.

Figure 8 shows the ethanol concentrations obtained in fermentations using *W. anomalus* M15 with different initial glucose concentrations in seaweed hydrolysates. For fermentations using synthetic brown seaweed hydrolysate, the ethanol concentration is observed to level off with the increase of initial glucose concentration (and the total sugar concentration as well), suggesting other factors (likely to be mannitol inhibitory affect) limits ethanol synthesis. It was reported that 10 g/L mannitol reduced the ethanol synthesis yield by a factor of 12.7% in fermentations using *S. cerevisiae* (Wei et al., 2021). For fermentations using synthetic red seaweed and green seaweed hydrolysates, the relationships are linear, indicating initial glucose concentration is the only factor that limits the final ethanol concentration. The data obtained from fermentations using actual green seaweed hydrolysate (*Ulva spp.*) were also plotted into Figure 8 (this study and Greetham et al., 2020). In general, the relationship between ethanol concentration and initial glucose concentration also fit the trend that was obtained using synthetic green seaweed. Although using synthetic seaweed hydrolysate media may not reflect the actual fermentation profiles of actual seaweed hydrolysate, it is sufficient for the exploration of the strain’s potential in biofuel synthesis. In the scenario that it is challenging to achieve high sugar content *via* the hydrolysate and subsequent concentration strategies, synthetic seaweed hydrolysate is a viable alternative for the characterisation of the microorganisms. In the fed-batch fermentation with synthetic green seaweed hydrolysate, similar ethanol concentrations (71.8 g/L and 73.8 g/L) were obtained from 162.4 g/L initial glucose, regardless of whether seawater or deionised water was used. It also proved that at an ethanol concentration of at least 73.8 g/L, strain *W. anomalus* M15 performed normally as it would at low sugar and alcohol concentrations. These results indicated that the limitation of bioethanol production from seaweed lies mainly on the ability to provide a high sugar content in the fermentation medium. If a suitable seaweed hydrolysate with high sugar content could be provided, the ethanol production could exceed 70 g/L, or possibly exceed 90 g/L as suggested in fermentation using the adaptation strain of *W. anomalus* M15 (Figure 7).

In this study, high salt content was obtained in the hydrolysis of green seaweed *U. linza*. The salinity proved challenging when concentrating the sugar content of the seaweed hydrolysate. The high salt content could have come from three possible sources: 1) the high salt content within the seaweed; 2) fine sand mixed with green seaweed; 3) salt formed *via* acid pre-treatment and the following neutralisation using alkali. Several papers reported that seaweed contains high content of ash, for *U. linza*, it is reported to be in the range of 21.5–30.2% (Greetham et al., 2018). In seaweed, salt is an important component to balance osmotic stress in the growing environment. Green seaweed is delicate and does not exhibit the structural integrity required for growth to match that of brown seaweed. Fine sand adhered or ingrained in the green seaweed harvested was observed during processing. In this study, seaweed was processed by rinsing in fresh water, separation of sand and detritus was conducted by a combination of flotation and sieving after drying. This was combined with cold filtration and ion exchange post hydrolysis and concentration to remove salt, however results were unsatisfactory.

Separating salt from marine algal biomass will likely be a challenging task for bioethanol production. In order to reduce the formation of salt *via* the addition of acid in the pre-treatment step, utilizing a lower diluted acid concentration, such as 1% (w/w) is recommended (Greetham et al., 2020). Higher acid concentration may lead to a slightly improved hydrolysis yield, but the removal of salt from the sugar solution will be costly. Likewise, the utilization of seawater during pretreatment of the hydrolysate yielded a higher hydrolysis conversion, although may inhibit the final ethanol production yield. The reduction of sugar content during the ion exchange step was unexpected, especially as the reduction only occurred in the hydrolysate obtained using seawater. Crystallised salt particles were removed by filtration prior to ion exchange. It is possible that a certain amount of sugar crystallised and was removed together with the salt. In any case, utilizing a high salt tolerance strain will be important, in which marine yeast could make a significant contribution.

Besides seaweed, many types of non-food biomass have been investigated for fermentative biofuel production, such as wheat straw (Kumar et al., 2020), sugarcane bagasse (Kumar et al., 2020), *Miscanthus* (Turner et al., 2021), willow (Stephenson et al., 2010) and food waste (Zhang et al., 2020). Comparison to land based biomass, marine biomass does not occupy agriculture land and does not consume fresh water resource. Therefore algal biofuel is considered as a greener, more advanced generation of biofuels. Nonetheless, a common challenge in using non-food biomass for biofuel fermentation is to achieve high sugar content economically in the biomass hydrolysate. High titer of bioethanol fermentation could be realised once sufficient sugar is obtained in biomass hydrolysate.

## 5 Conclusion

In this study, the glucose, xylose and ethanol tolerances of a marine yeast *Wickerhamomyces anomalus* M15 were determined to be 600, 150 and 250 g/L, respectively. Fermentations with *W. anomalus* M15 using simulated concentrated seaweed hydrolysates resulted in 5.8, 45.0 and 19.9 g/L ethanol from brown, green and red seaweed based media, indicating green seaweed *Ulva* spp. is the best substrate in terms of bioethanol production using *W. anomalus* M15. Fed-batch fermentations with both seawater and fresh water were carried out, leading to an ethanol concentration of 73 g/L, with no considerable differences between fresh and sea water. Concentrating sugars in the actual seaweed hydrolysate was challenging, mainly due to high salt content in the seaweed hydrolysate. Around 15.5 g/L bioethanol was produced from *Ulva* spp*.* hydrolysate harvested from United Kingdom shores. A promising adapted strain of *W. anomalus* M15 was isolated, which produced 92.7 g/L ethanol from 200 g/L glucose, indicating its potential to be used in commercial bioethanol production.

## Data Availability

The original contributions presented in the study are included in the article/Supplementary Material, further inquiries can be directed to the corresponding author.
