# Supplementary material for: The characterisation of Wickerhamomyces anomalus M15, a highly tolerant yeast for bioethanol production using seaweed derived medium
Source: Front Bioeng Biotechnol. 2022 Oct 13;10:1028185. doi: 10.3389/fbioe.2022.1028185 (PMC9608644; doi:10.3389/fbioe.2022.1028185)
Supplement: Supplementary file 1 [file Table1.DOCX]

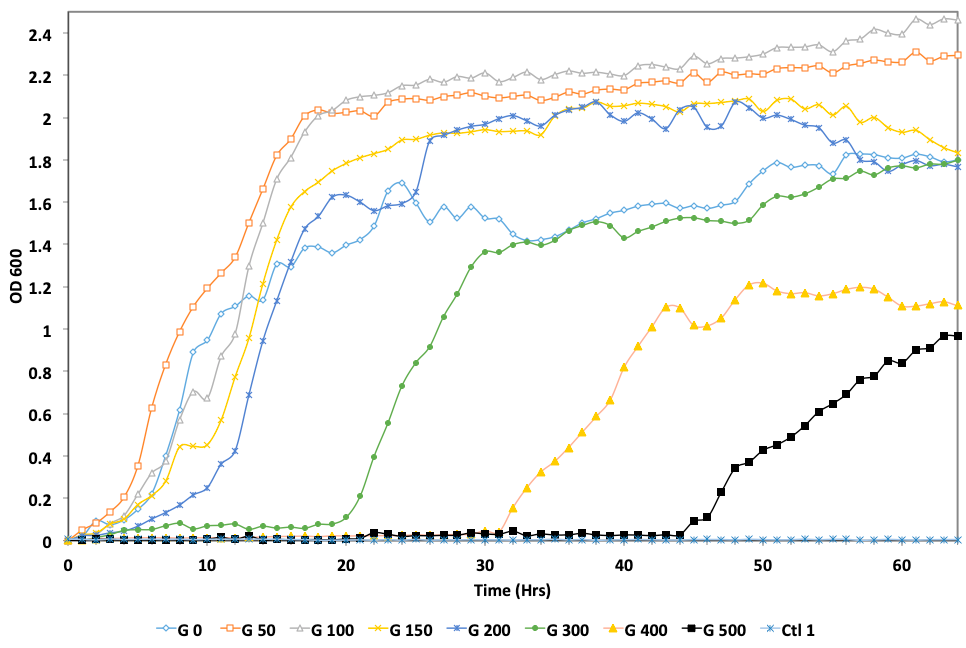


Figure Appendix A1: Growth profiles of *W. anomalus* M15 at different initial glucose concentration. G 0, 0 g/L glucose; G 50, 50 g/L glucose; G 100, 100 g/L glucose; G 150, 150 g/L glucose; G 200, 200 g/L glucose; G 300, 300 g/L glucose; G 400, 400 g/L glucose; G 500, 500 g/L glucose; Ctl 1, (control) no inoculation.


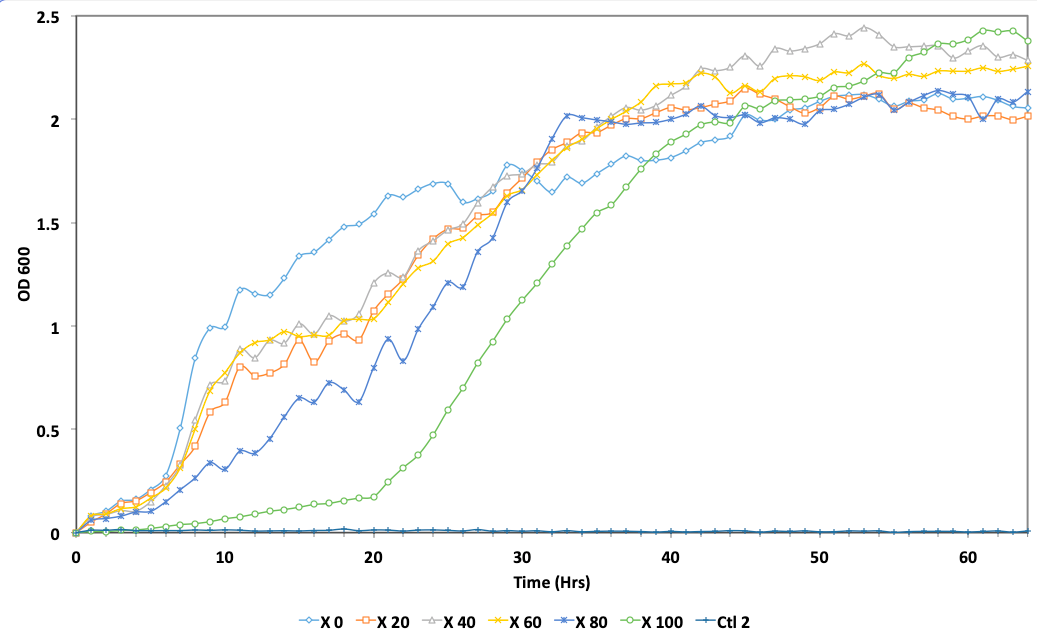


Figure Appendix A2: Growth profiles of *W. anomalus* M15 at different initial xylose concentration. X 0, 0 g/L xylose; X 20, 20 g/L xylose; X 40, 40 g/L xylose; X 60, 60 g/L xylose; X 80, 80 g/L xylose; X 100, 100 g/L xylose; Ctl 2, (control) no inoculation.


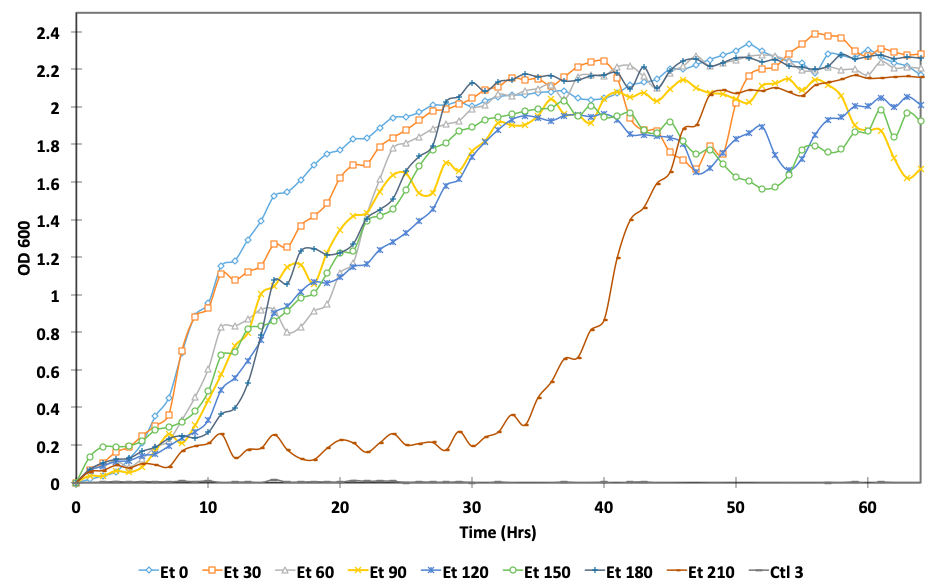


Figure Appendix A3: Growth profiles of *W. anomalus* M15 at different initial ethanol concentration. Et 0, 0 g/L ethanol; Et 30, 30 g/L ethanol; Et 60, 60 g/L ethanol; Et 90, 90 g/L ethanol; Et 120, 120 g/L ethanol; Et 150, 150 g/L ethanol; Et 180, 180 g/L ethanol; Et 210, 210 g/L ethanol; Ctl 3, (control) no inoculation.


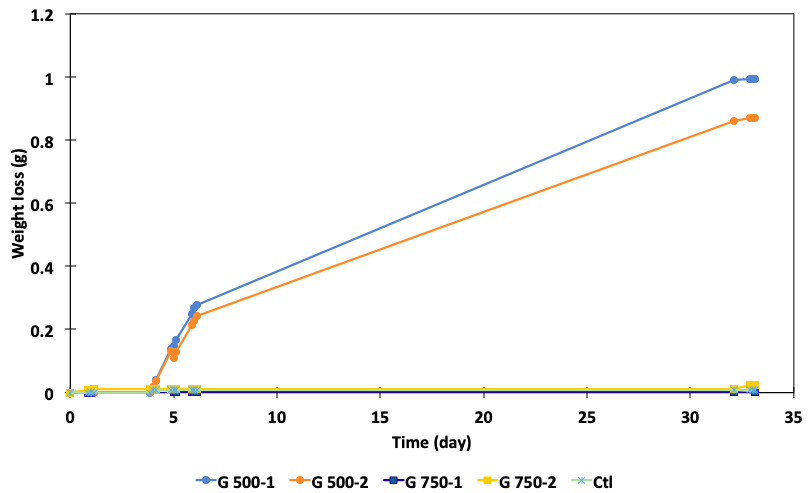


Figure Appendix A4: Growth profiles of *W. anomalus* M15 at different initial glucose concentration over a long period. G 500-1, 500 g/L glucose, trial 1; G 500-2, 500 g/L glucose, trial 2; G 750-1, 750 g/L glucose, trial 1; G 750-2, 750 g/L glucose, trial 2; Ctl, (control) no inoculation.
